# Equivalent gain-of-function variants in *KCNK3* and *KCNK9* and their contribution to distinct TASK K2P channelopathies

**DOI:** 10.1085/jgp.202613989

**Published:** 2026-08-06

**Authors:** Kate M. Crowther, Thibault R.H. Jouen-Tachoire, Peter Proks, Peter Rory Hall, Emma L. Veale, Janina Sörmann, Karin E.J. Rödström, Thomas Müller, Saskia B. Wortmann, Nina Barisic, Natalie Hauser, Vincenzo Salpietro, RaeLynn Forsyth, Linford Williams, Nora Derrabi, Carlos A. Bacino, Jill A. Rosenfeld, Henry Houlden, Simon Newstead, Caroline F. Wright, James Fasham, Alistair A. Mathie, Reza Maroofian, Stephen J. Tucker

**Affiliations:** 1 Clarendon Laboratory, Department of Physics, University of Oxford, Oxford, UK; 2 Kavli Institute for Nanoscience Discovery, University of Oxford, Oxford, UK; 3 Department of Pharmacology, University of Oxford, Oxford, UK; 4 Department of Biochemistry, University of Oxford, Oxford, UK; 5 Department of Integrative Structural and Computational Biology, The Scripps Research Institute, La Jolla, CA, USA; 6 Medway School of Pharmacy, Universities of Greenwich and Kent at Medway, Chatham Maritime, UK; 7 School of Life Sciences, University of Westminster, London, UK; 8 Bayer AG, Research & Development, Pharmaceuticals, Wuppertal, Germany; 9 University Children’s Hospital, Paracelsus Medical University, Salzburg, Austria; 10 University Hospital Centre Split, School of Medicine, University of Zagreb, Zagreb, Croatia; 11 Department of Pediatrics, Inova Fairfax Hospital, Falls Church, VA, USA; 12 Department of Neuromuscular Diseases, UCL Queen Square Institute of Neurology, London, UK; 13 Department of Pediatrics, UPMC Children’s Hospital of Pittsburgh, Pittsburgh, PA, USA; 14 Department of Anatomic Pathology, CHU Ibn Rochd Hospital, Casablanca, Morocco; 15 Department of Molecular and Human Genetics, Baylor College of Medicine, Houston, TX, USA; 16 Baylor Genetics Laboratories, Houston, TX, USA; 17 Department of Clinical and Biomedical Sciences, University of Exeter Medical School, Exeter, UK; 18 Peninsula Clinical Genetics Service, Royal Devon & Exeter Hospital (Heavitree), Exeter, UK; 19 OXION Initiative in Ion Channels & Disease, University of Oxford, Oxford, UK

## Abstract

Gain-of-function (GoF) missense variants in the two-pore domain (K2P) K^+^ channel TASK-1 (*KCNK3*) result in developmental delay with sleep apnea (DDSA), a neurodevelopmental channelopathy, while loss-of-function (LoF) variants cause pulmonary arterial hypertension. However, for the related TASK-3 channel (*KCNK9*), both LoF and GoF variants underlie a distinct neurodevelopmental disorder, *KCNK9* imprinting syndrome (KIS). The relationship between genotype and phenotype in these disorders is further complicated because TASK-1 and TASK-3 can co-assemble into heteromeric channels with distinct functional properties. Here, we report additional patients with missense variants in *KCNK3* and *KCNK9* and investigate the effect of four novel genetic variants on the functional properties of homomeric and heteromeric TASK channels. Interestingly, two of these new pathogenic GoF variants (R131H and L122V) are found in both TASK-1 and TASK-3 and have equivalent functional effects on heteromeric TASK-1/TASK-3 channel activity, yet result in different clinical phenotypes. We have also determined a cryo-EM structure for the pathogenic L122V mutant TASK-3 channel, which suggests that subtle changes in gating and permeation within the inner cavity are responsible for its activatory effect. Overall, these results highlight the dominant role that homomeric TASK channels likely play in defining their associated channelopathies as well as the complexity of interpreting K^+^ channel dysfunction in pathophysiology.

## Teaser

In this study, Crowther and colleagues report new disease-causing variants in the TASK-1 and TASK-3 potassium channels responsible for two different neurodevelopmental disorders. They also examine the structural and functional properties of these variants and examine their effects when incorporated into heteromeric TASK-1/TASK-3 channels.

## Introduction

Genetic variants that affect the function of K2P K^+^ channels are now increasingly associated with a range of diseases, including diabetes, nephropathy, migraine, sleep apnea, hypertension, and other cardiac disorders, as well as a range of neurodevelopmental disorders (Lee et al., 2021). However, despite the pressing need, there are few effective treatments for these complex conditions, and their underlying mechanisms are often not well understood.

K2P channels represent a structurally distinct subfamily of K^+^ channels. In addition to their distinct extracellular Cap domain, each subunit contains four transmembrane helices (M1–M4) and two pore domains (P1 and P2). Two subunits therefore assemble as a “dimer of dimers” to create a pseudotetrameric channel that is similar to other classical K^+^ channel pores (Enyedi and Czirjak, 2010; Niemeyer et al., 2016). There are 15 different human K2P (*KCNK*) channels, and members of this subfamily of K^+^ channels are often referred to as simple “leak” conductances involved in establishing the resting membrane potential, but it is now clear they exhibit complex patterns of dynamic regulation by diverse stimuli, including many G-protein–coupled receptor (GPCR)-associated regulatory pathways (Enyedi and Czirjak, 2010; Niemeyer et al., 2016). Understanding K2P channel dysfunction in the disease state is therefore critical for a clearer insight into these rare channelopathies and to exploit the therapeutic potential of K2P channels in other more common disease states (Bagal et al., 2013; Mathie et al., 2021).

**Figure S1.**
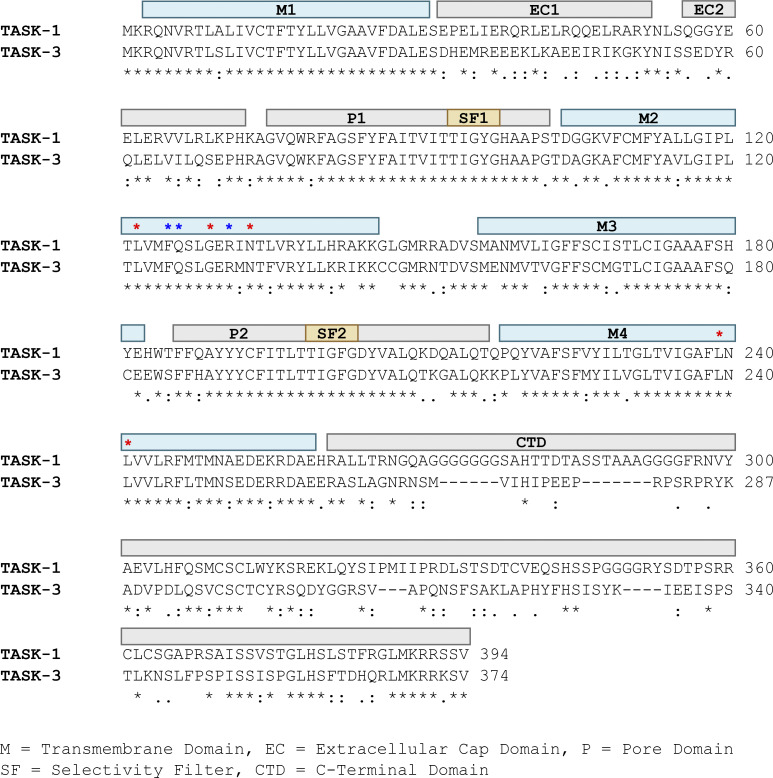
**Homology between TASK-1 and TASK-3.** An amino acid alignment between the sequences of TASK-1 (*KCNK3*) and TASK-3 (*KCNK9*) reveals the high degree of homology between the two channel subunits. Overall, the sequence identity is 54% with almost 80% identity within the pore-forming transmembrane regions. The extent of the homology means that within the TM/Pore region the equivalent amino acid numbering is identical and does not deviate except within the CTD. The various transmembrane regions (M1–M4) are indicated by blue bars above the sequence along with the Extracellular Cap domain helices and the selectivity filters. The position of the variants is indicated by asterisks (red = existing variant, blue = new variant described in this study). This clearly demonstrates that M2 is a hotspot for pathogenic GoF variants in TASK-1.

*KCNK3* and *KCNK9* encode the TASK-1 and TASK-3 K2P channels, respectively. Although separate genes with distinct tissue-specific patterns of expression, they share almost 80% amino acid identity within the main pore-forming transmembrane regions (Fig. S1). Consequently, they exhibit significant structural and functional similarity (Rodstrom et al., 2020; Lin et al., 2024; Hall et al., 2025), and in those cell types where they are coexpressed, they can co-assemble into heteromeric TASK-1/TASK-3 channels, which possess distinct functional properties (Berg et al., 2004; Kim et al., 2009; Bohnen et al., 2017; Rinné et al., 2024).

TASK-1 (*KCNK3*) is broadly expressed in many cells and tissues involved in the control of breathing and ventilation, including the carotid body, lung, heart, and pulmonary artery smooth muscle, as well as many ventilatory-associated chemosensitive regions of the brain, hypoglossal motor neurons, and spinal cord motor neurons (Trapp et al., 2008; Bayliss et al., 2015; Lin et al., 2025). By contrast, TASK-3 (*KCNK9*) expression shows a higher degree of specialization within the central nervous system (CNS). However, their patterns of expression do overlap in some cell types; for example, both subunits are coexpressed in carotid body glomus cells (Buckler et al., 2000; Buckler, 2015), and heteromeric TASK-1/TASK-3 channels in these cells are proposed to form the major oxygen-sensitive background K^+^ channel, which plays a critical role in the regulation of respiration. Both TASK-1 and TASK-3 are also a target for volatile anesthetics (Conway and Cotten, 2012; Luethy et al., 2017), and given their key role in ventilation and respiratory control, inhibitors that target TASK-1 have been developed for the treatment of sleep apnea with improvements observed in a subset of patients, where topical application of the inhibitor in the upper airway was achieved via nasal spray (Osman et al., 2024).

Given their physiological role and expression profile, it was perhaps not surprising that inherited monoallelic loss-of-function (LoF) variants in *KCNK3* have been shown to be associated with a form of pulmonary arterial hypertension (Ma et al., 2013; Olschewski et al., 2017; Jouen-Tachoire et al., 2021). Furthermore, we have also recently shown that *de novo* heterozygous missense gain-of-function (GoF) variants underlie a novel neurodevelopmental disorder (developmental delay with sleep apnea [DDSA]), where all affected individuals also have sleep apnea (Kaplanis et al., 2020; Sörmann et al., 2022). Structural analysis reveals the majority of these mutations cluster in the M2 and M4 transmembrane helices close to an intersection between which forms the “X-gate,” a structural motif analogous to the classical bundle-crossing gate in other K^+^ channels (Rodstrom et al., 2020; Sörmann et al., 2022). All of these variants affect channel gating and produce TASK-1 channels with a higher intrinsic single-channel open probability (*P*_*o*_) combined with a markedly reduced sensitivity to inhibition by Gq-protein–coupled receptor (GqPCR) pathways (Sörmann et al., 2022). We have recently shown that this defect in GPCR inhibition is directly coupled to their higher intrinsic *P*_*o*_ and the state-dependent inhibition of TASK channels by PLC-generated diacylglycerol (Jouen-Tachoire et al., 2026).

Missense variants in TASK-3 (*KCNK9*) are associated with a different neurodevelopmental disorder, “*KCNK9* imprinting syndrome” (KIS), previously known as Birk-Barel syndrome (Barel et al., 2008; Graham et al., 2016). Typically, KIS-associated mutations produce a LoF in TASK-3, but more recently, GoF mutations have also been reported in individuals that present similar phenotypes (Cousin et al., 2022). Sleep apnea has also been reported in some KIS patients (Cousin et al., 2022) but is not a universal feature, unlike its defining phenotype in DDSA. It remains unclear why both LoF and GoF variants in *KCNK9* result in the same phenotype, i.e., KIS. This complex etiology may be due to genomic imprinting of *KCNK9*, which has not been reported for *KCNK3* or any other K2P channels, as well as the mosaicism associated with the *de novo* origin of these variants (Luedi et al., 2007; Cooper et al., 2020; Cousin et al., 2022). Another complicating factor may be their ability to form heteromeric channels as the relative contribution of defects in homomeric vs. heteromeric channels to these different disorders also remains unclear.

In this study, we identify six new affected individuals with GoF mutations in TASK-1, which display a DDSA phenotype, and these include three novel missense variants in *KCNK3* (F125S, Q126E, and R131H). We also identify a novel missense variant in *KCNK9* (L122V) associated with KIS and solve a cryo-EM structure of this mutant channel. Overall, the structural and functional characterization of these different mutations provide important insights into the relative contributions of both homomeric and heteromeric TASK channels in the development of TASK-related disorders.

## Materials and methods

### Patient identification, genetic and clinical investigation, and ethical approval

Using the GeneMatcher platform (Kaplanis et al., 2020), Genomics England data derived from the 100,000 genomes project, and extensive international data sharing, we identified seven families. A uniform clinical pro forma was distributed to collect clinical details. Parents or legal guardians of all affected individuals provided informed consent for the publication of clinical information and genetic data, in accordance with the Declaration of Helsinki. The study was approved by the relevant local Ethics Committees, including University College London (for Integrated Research Application System Project ID: 310045), while the original projects (Kaplanis et al., 2020; Sörmann et al., 2022) have UK Research Ethics Committee approval (10/H0305/83, granted by the Cambridge South REC, and GEN/284/12, granted by the Republic of Ireland REC). Trio exome or genome sequencing, as well as patient-only sequencing where applicable, was performed on DNA extracted from blood-derived leukocytes. Data analysis and variant filtration were conducted using standard pipelines, and Sanger sequencing was used for segregation analysis when indicated.

### Electrophysiology

Whole-cell currents were measured using two-electrode voltage clamp (TEVC) recordings of *Xenopus laevis* oocytes microinjected with the relevant mRNA. This was done as previously described in accordance with local ethical and regulatory requirements (Sörmann et al., 2022). Briefly, the WT human TASK-1 gene (*KCNK3*) and human TASK-3 gene (*KCNK9*) were subcloned into pFAW. Mutations were introduced by site-directed mutagenesis and confirmed by sequencing. mRNA was transcribed using the T7 mScript Standard mRNA Production System. Each oocyte was then injected with 2 ng of RNA and incubated for 20–24 h at 17.5°C. Recordings were performed in ND96 buffer at pH 7.4 (96 mM NaCl, 2 mM KCl, 2 mM MgCl_2_, 1.8 mM CaCl_2_, and 5 mM HEPES). Currents were recorded using a 400 ms voltage step protocol from a holding potential of −80 mV delivered in 10 mV increments between −120 mV and +50 mV and 800 ms ramp protocols from −120 to +50 mV. All recorded traces were analyzed using Clampfit, and graphs were plotted using Origin 2021. Single-channel currents in cell-attached patches were recorded under quasi-symmetrical potassium concentrations with 140 mM KCl solution containing 10 mM HEPES (pH 7.4 with KOH) in the pipette. Data were filtered at 2 or 5 kHz and recorded at a 200 kHz sampling rate with program Clampex on an Axopatch 200B amplifier. Data analysis was performed using Clampfit. Channel open probability (*P*_*o*_) was determined from single-channel recordings with 1–4-min duration, which exhibited only one main open level. Numbers of individual recordings (*n*) are quoted for each value along with the test used for any statistical comparison (one-way ANOVA with Dunnett’s multiple comparisons test). ns, not significant; * = P < 0.05; ** = P < 0.01; *** = P < 0.001.

### Cryo-EM sample preparation and collection

TASK-3 L122V protein was expressed and purified using the method previously described for WT TASK-3 (Hall et al., 2025). Samples of TASK-3 L122V (4.0 mg/ml) were then applied onto glow-discharged AuFlat 300 1.2/1.3 gold grids (Molecular Dimensions). The grids were blotted for 4–5 s at 100% humidity and 4°C and then vitrified in liquid ethane using a Vitrobot Mark IV (Thermo Fisher Scientific). Data were collected on a Titan Krios G3 (FEI) operating at 300 kV, equipped with a BioQuantum imaging filter (Gatan) and a K3 direct detection camera (Gatan). Images were acquired at a magnification of 105,000×, corresponding to a physical pixel size of 0.832 Å with a total dose rate of 43.9 e^-^/Å^2^. A total of 13,035 initial movies were collected.

### Image processing

All processing was performed in cryoSPARC (Punjani et al., 2017) unless stated otherwise. Movies were imported and underwent motion correction and patch contrast transfer function estimation. A subset of 378 micrographs was selected for picking with a particle diameter of 125 Å. The initially extracted particles underwent two rounds of 2D classification and *ab initio* reconstruction (*n* = 2), which yielded a volume used to generate templates for particle picking.

The TASK-3 L122V templates were used for particle repicking from the full dataset with a diameter of 125 Å, resulting in the extraction of 8,381,007 particles with box size of 256 px, Fourier cropped to 128 px. These particles underwent four rounds of 2D classification during which junk particles were removed, leaving 1,718,239 particles. The particles were re-extracted with a full 256 px box size, prior to two more rounds of 2D classification. Two rounds of five *ab initio* classes and heterogenous refinement in C1 and C2 symmetry were completed, leading to a particle stack of 332,112 particles. Homogenous refinement, nonuniform (NU) refinement, and reference-based motion correction in C2 produced a 2.90 Å map. Polished particles were processed with cryosieve (Zhu et al., 2023), and the particle stacks were reimported into cryoSPARC. To identify the highest resolution stack, each stack was subjected to one round of 2D classification, *ab initio* reconstruction in C1, followed by homogeneous and NU refinements in C2. The final particle set (211,335 particles) produced a map with a resolution of 2.83 Å, based on the FSC = 0.143 criterion.

### Model building and refinement

WT TASK-3 (Protein Data Bank [PDB] ID: 9G9V) was used as the initial model to build TASK-3 L122V. The structure was manually mutated and adjusted within the map using Coot (Emsley et al., 2010). The model was further refined iteratively, first through manual adjustments in Coot and then using phenix.real_space_refine (Adams et al., 2010). The refined model was subsequently processed with ISOLDE (Croll, 2018) through ChimeraX (Meng et al., 2023) to enhance their accuracy and underwent a final round of refinement in Phenix, with the ISOLDE-optimized models serving as references. Structural figures were generated using PyMOL (Schrödinger, LLC).

### Online supplemental material

**Figure S2.**
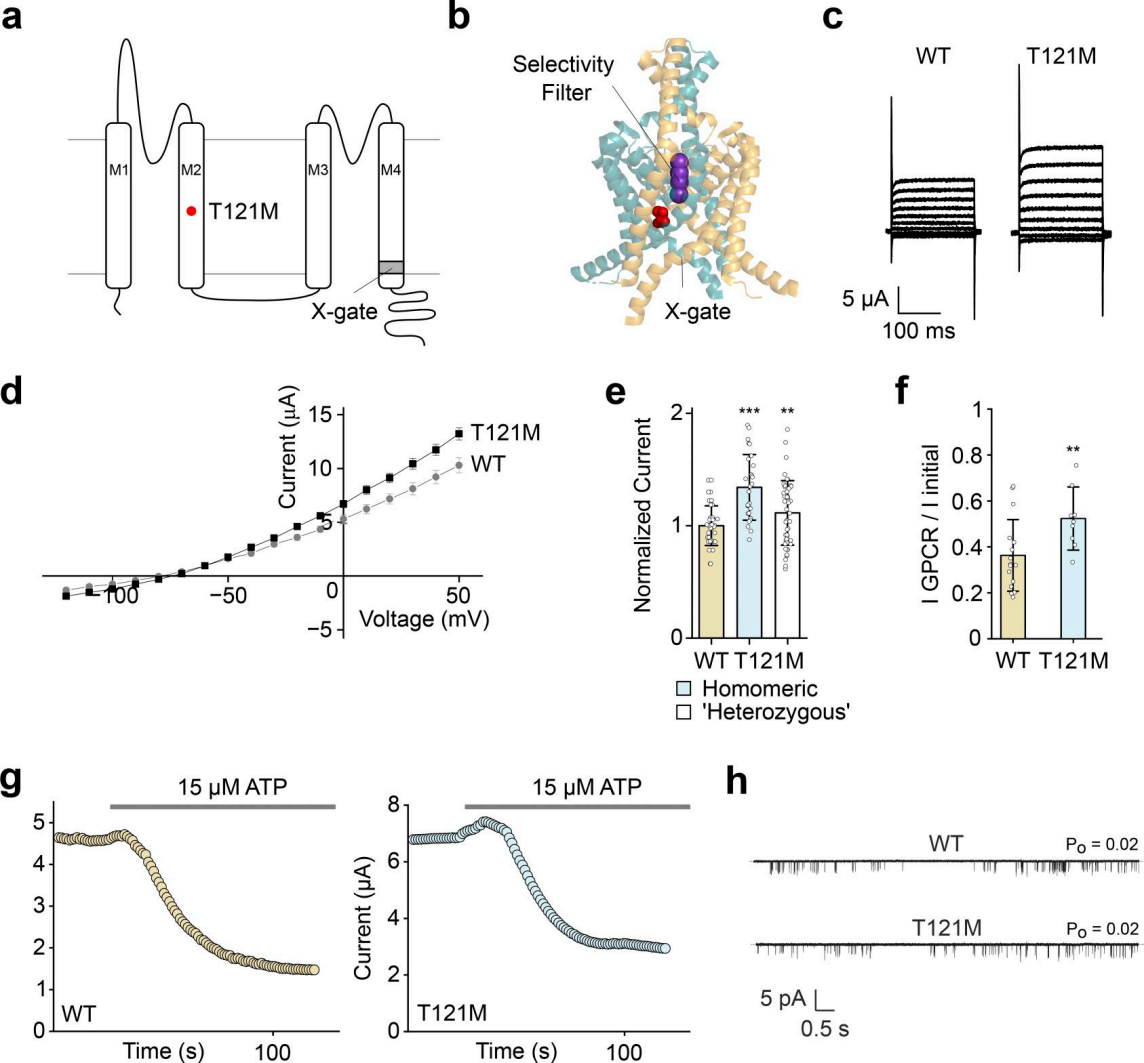
**Functional effects of the T121M mutation. (a)** 2D topological model of a TASK-1 subunit with the position of the T121M mutation labelled in red. The X-gate is labelled in dark grey. **(b)** TASK-1 crystal structure (PDB: 6RV2). Subunits are shown in teal and orange, respectively. The T121M mutation is shown as red spheres. **(c)** Representative TEVC recordings of WT TASK-1 and T121M mutant currents in response to voltage steps from −120 to +50 mV in 20 mV steps from a holding potential of −80 mV. **(d)** Current–voltage plot of WT TASK-1 (*n* = 12) and T121M (*n* = 16); data are presented as mean ± SEM. **(e)** Currents for homomeric and heterozygous T121M channels formed from 1:1 coexpression of WT TASK-1 and T121M normalized to WT current at +50 mV: WT (*n* = 27), T121M (*n* = 28, P = 1.5 × 10^−10^), and T121M-WT (*n* = 48, P = 5.3 × 10^−5^). Data are presented as mean ± SD, one-way ANOVA with Dunnett’s multiple comparisons test. **(f)** Residual current for homomeric T121M after addition of 15 μM ATP. WT TASK-1 (*n* = 15), and T121M (*n* = 9, P = 4.3 × 10^−5^); data are presented as mean ± SD, one-way ANOVA with Dunnett’s multiple comparisons test. ns, not significant; ** = P < 0.01; *** = P < 0.001. **(g)** Representative currents at +50 mV of WT TASK-1 and T121M channels, over time while adding 15 µM ATP. **(h)** Single-channel *P*_*o*_ values at −100 mV, WT (*n* = 8) and T121M (*n* = 8).

**Figure S3.**
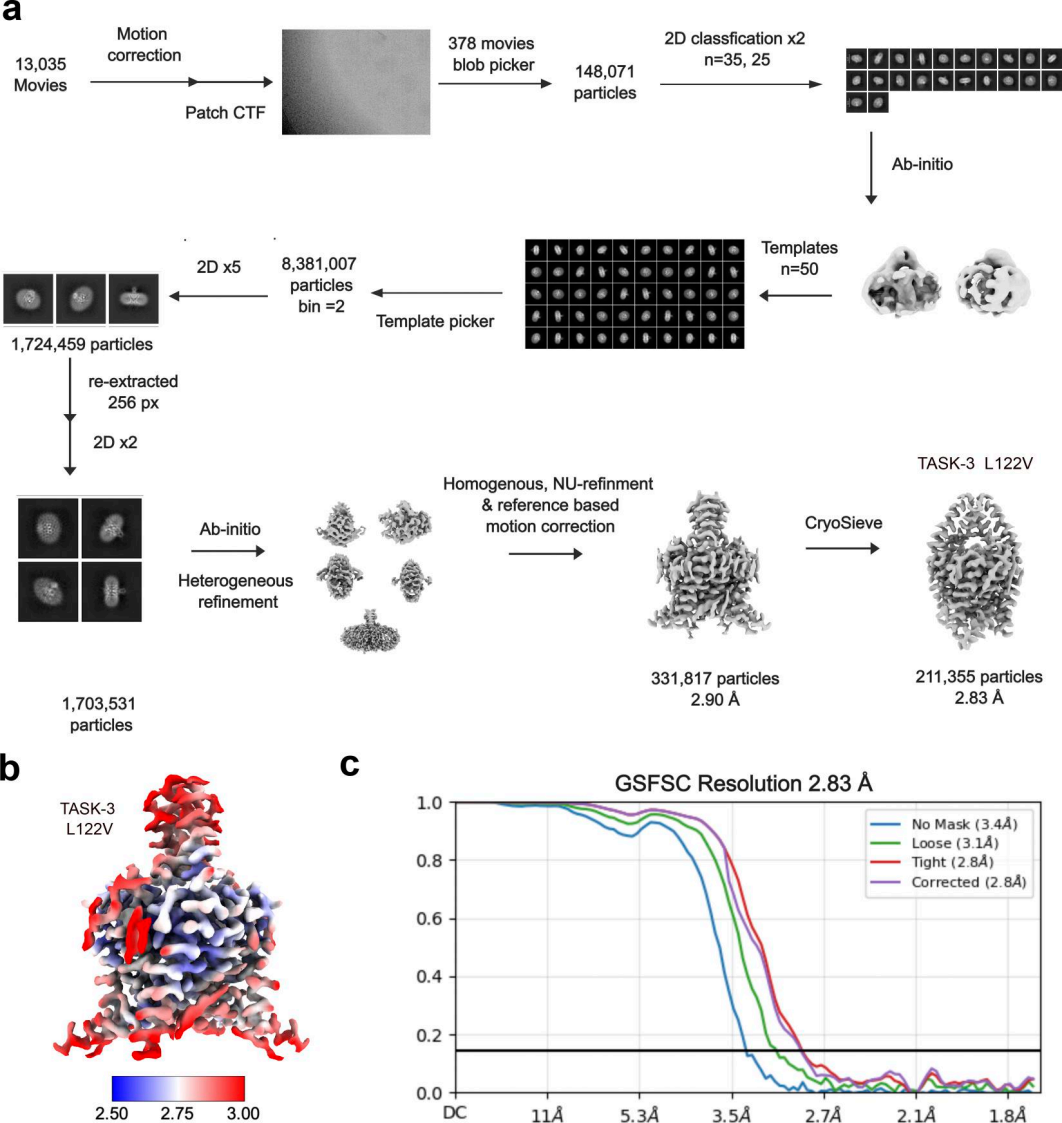
**Determination of cryo-EM Structure of the TASK-3 L122V mutation. (a)** Image processing workflow for TASK-3 L122V. **(b)** Local resolution of reconstructed map as determined within cryoSPARC. **(c)** Gold-standard FSC curve used for global-resolution estimates within cryoSPARC.

Fig. S1 shows an annotated alignment of the sequences of TASK-1 and TASK-3. Fig. S2 shows a summary of the functional properties of the TASK-1 T121M variant. Fig. S3 illustrates the image processing workflow and associated data for the structure of TASK-3 L122V. Table S1 summarizes the phenotype and anonymized clinical details of the new DDSA and KIS patients reported in this study. Table S2 summarizes the cryo-EM data collection, refinement, and validation statistics for the TASK-2 L122V structure reported in this study.

## Results

### Three novel DDSA variants in KCNK3

**Figure 1.**
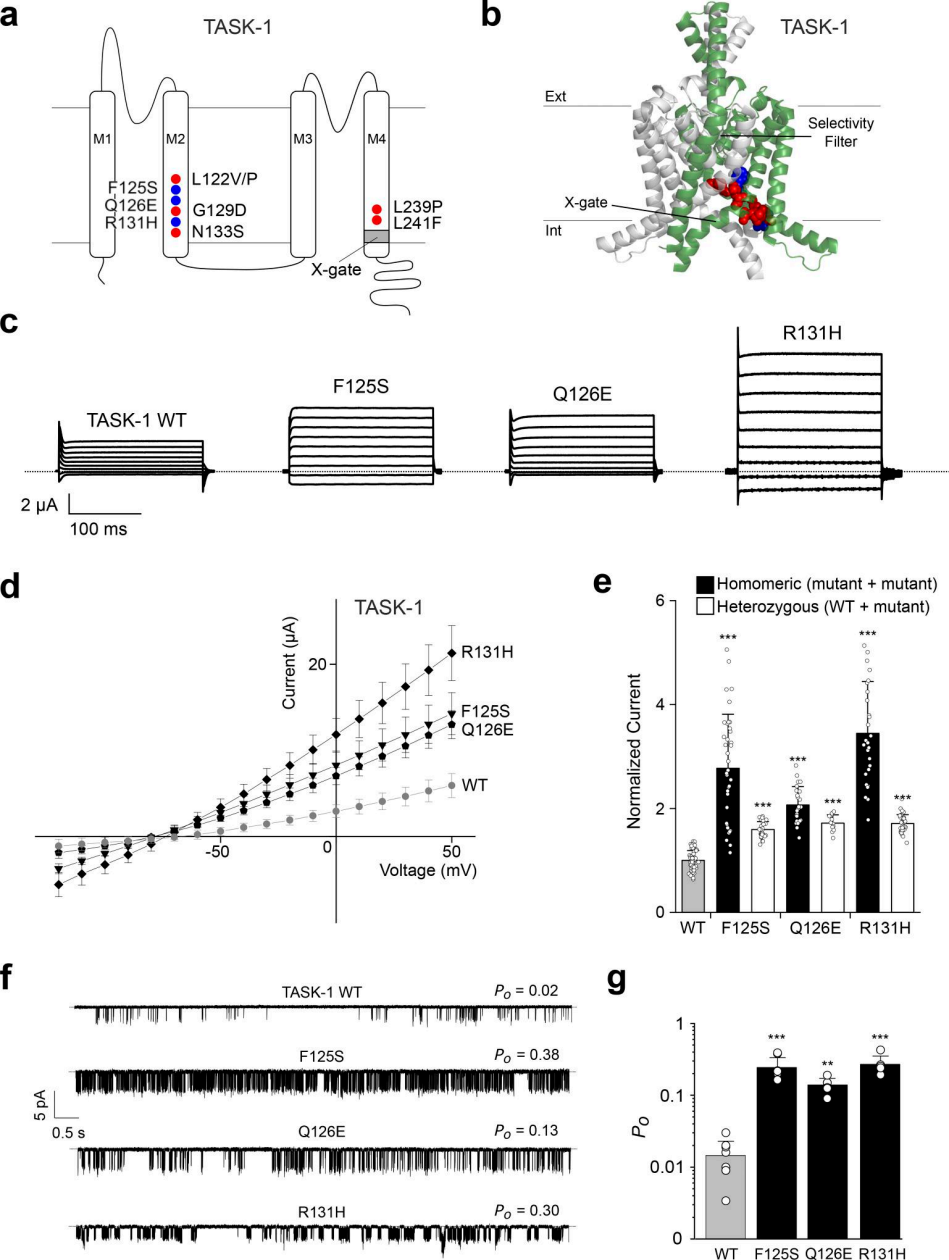
**Functional effects of new DDSA-associated variants. (a)** Transmembrane topology of TASK-1 shown with the position of the new and previously characterized DDSA variants labelled in blue and red, respectively. The position of the X-gate on M4 is also shown. **(b)** TASK-1 crystal structure (PDB: 6RV2) with the two subunits shown in green and grey, respectively. The new and previously characterized DDSA mutations are shown on one subunit as blue and red spheres, respectively. **(c)** Representative TEVC recordings of WT TASK-1 and DDSA mutant currents in response to voltage steps from −120 to +50 mV in 20 mV steps from a holding potential of −80 mV. **(d)** Current–voltage plot of WT TASK-1 (*n* = 8), F125S (*n* = 16), Q126E (*n* = 19), and R131H (*n* = 23); data are presented as mean ± SD. **(e)** Currents for homomeric DDSA mutants, and heterozygous channels formed from 1:1 coexpression of WT TASK-1 and DDSA mutants normalized to WT current at +50 mV: WT (*n* = 43), F125S (*n* = 26, P = 1.4 × 10^−22^), F125S-WT (*n* = 16, P = 4.4 × 10^−6^), Q126E (*n* = 22, P = 1.1 × 10^−14^), Q126E-WT (*n* = 12, P = 4.7 × 10^−6^), R131H (*n* = 17, P = 1.1 × 10^−55^), and R131H-WT (*n* = 23, P = 5.8 × 10^−8^); data are presented as mean ± SD All mutant currents differ from WT (one-way ANOVA with Dunnett’s multiple comparisons test). **(f)** Representative single-channel recordings of new DDSA mutants at a holding potential of −160 mV. **(g)** Single-channel *P*_*o*_ for each mutant. WT (*n* = 8), F125S (*n* = 8, P = 5.4 × 10^−7^), Q126E (*n* = 6, P = 0.0037), and R131H (*n* = 6, P = 3.2 × 10^−7^). All mutant *P*_*o*_ values differ from that for WT (one-way ANOVA with Dunnett’s multiple comparisons test). ns, not significant; ** = P < 0.01; *** = P < 0.001.

Our previous study identified nine individuals with DDSA (Sörmann et al., 2022), each heterozygous for one of six *de novo* missense variants in *KCNK3*. Since that original study in 2022, we have now identified another three DDSA patients with similar *de novo* GoF mutations in *KCNK3,* including two with the recurrent N133S variant and one with the L241F variant. In addition, we have identified three further affected individuals with novel heterozygous missense variants in *KCNK3* who all match the reported DDSA neurodevelopmental phenotype (Table S1). Interestingly, the three new variants (F125S, Q126E, and R131H) all cluster in the M2 transmembrane helix adjacent to several previously characterized DDSA variants (Fig. 1, a and b). The location of these novel variants alongside many others within M2 indicates that this particular region of the transmembrane helix clearly represents a major hotspot for pathogenic mutations and is consistent with the proposed role for the TM helices and the X-gate in channel gating (Rodstrom et al., 2020; Sörmann et al., 2022; Hall et al., 2025). It is also consistent with the reported effects of other mutations within this region on K2P channel activity (Ben Soussia et al., 2019).

### Novel DDSA variants also produce GoF channels with impaired GPCR sensitivity

All existing DDSA variants are associated with a GqPCR-insensitive GoF effect on TASK-1 channel activity. Therefore, to confirm these new DDSA variants also fit this profile, we next examined their functional activity. Whole-cell K^+^ currents of WT and mutant TASK-1 channels were measured by TEVC after expression in *Xenopus* oocytes. These results show that all three variants produced markedly larger currents than WT TASK-1 (Fig. 1, c and d).

K2P channels assemble as dimers, and each of the new DDSA patients are also heterozygous for these *KCNK3* variants. To better replicate this, we therefore also measured average whole-cell currents from oocytes co-injected with equal amounts of WT and mutant TASK-1 RNA. Similar to the results obtained with the previous DDSA variants, we found that all three novel variants also increased these “heterozygous” currents by ∼50% (Fig. 1 e).

To confirm that these increases are due to an increase in the intrinsic activity of the channel rather than just an increase in trafficking to the cell surface, we also measured single-channel currents of homomeric WT and mutant TASK-1 channels and found that all three variants resulted in a marked (∼10-fold) increase in channel open probability (Fig. 1, f and g). A modest (∼20%) increase in single-channel conductance was also observed for the F125S and Q126E variants, but this was not large enough to account for their increased whole-cell currents (>250%).

**Figure 2.**
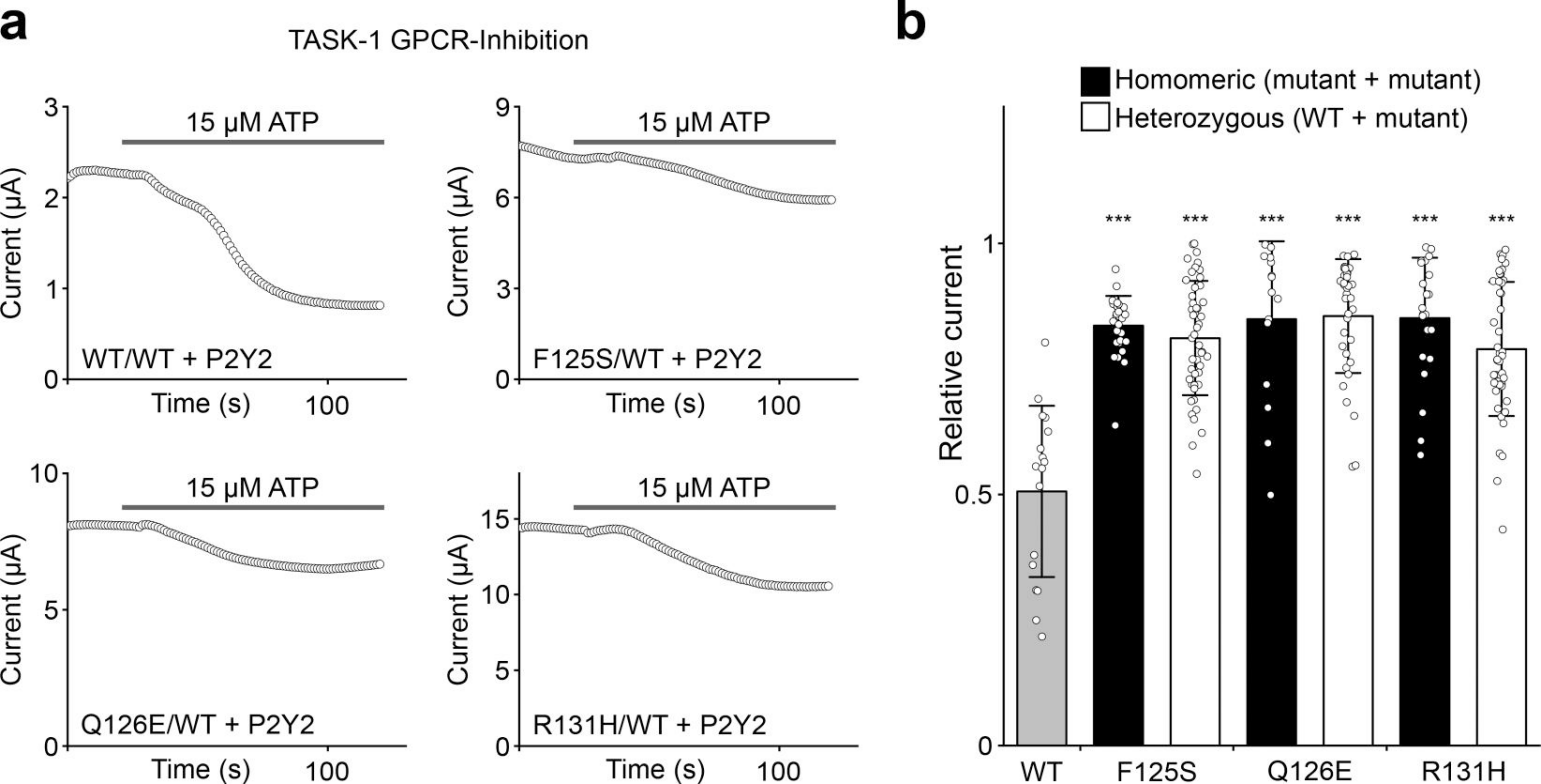
**GqPCR-mediated inhibition is reduced by DDSA-associated mutations. (a)** Representative currents at +50 mV of homomeric WT TASK-1 channels (WT/WT) and heterozygous channels from coexpressed WT and mutant subunits coexpressed with the P2Y2 receptor. Currents were recorded over time with 15 µM ATP applied extracellularly where indicated. This concentration produces ∼50% inhibition of WT TASK-1. **(b)** Residual current for homozygous mutants and mutants coexpressed 1:1 with WT TASK-1 after addition of 15 μM ATP. WT TASK-1 (*n* = 17), F125S (*n* = 28, P = 2.7 × 10^−15^), F125S/WT (*n* = 48, P = 4.4 × 10^−16^), Q126E (*n* = 15, P = 8.6 × 10^−13^), Q126E/WT (*n* = 36, P = 1.4 × 10^−18^), R131H (*n* = 22, P = 3.1 × 10^−15^), and R131H/WT (*n* = 45, P = 2.1 × 10^−13^); data are presented as mean ± SD. All mutant currents differ from WT (one-way ANOVA with Dunnett’s multiple comparisons test). ns, not significant; *** = P < 0.001.

WT TASK-1 channels are sensitive to inhibition by GqPCR mediated pathways, and the known DDSA variants are characterized by a dramatic reduction in this sensitivity that exacerbates their intrinsic GoF effect by uncoupling them from their regulatory pathways (Sörmann et al., 2022). We therefore also examined the GqPCR sensitivity of these new variants and found that whereas ATP-induced stimulation of a coexpressed P2Y2 receptor results in ∼50% inhibition of WT TASK-1 currents, all three variants exhibited a markedly reduced response with only 15–20% inhibition observed in identical conditions. This effect was seen in both homomeric and heterozygous TASK-1 mutant channels (Fig. 2). Thus, although the inheritance of the Q126E variant is unknown, its clear GoF effect as a heterozygous variant suggests it may also be of *de novo* origin, but this would need to be confirmed.

Together, these results confirm that these three novel *KCNK3* variants also fit the same functional profile as other DDSA variants by producing channels with an increased activity that is markedly less sensitive to inhibition via GqPCR-coupled signalling pathways. These functional defects will therefore have profound effects on the electrical activity of cells in which *KCNK3* is expressed, especially during development.

An additional inherited variant in *KCNK3* was also identified in 3-year-old child with behavioral problems, later diagnosed as an autism spectrum-like disorder and speech difficulties, but without sleep apnea. Interestingly, this variant (T121M) is also located in the M2 helix adjacent to the pathogenic L122V variant associated with DDSA. However, functional analysis revealed the T121M variant to behave similarly to WT TASK-1, producing only slightly elevated levels of whole-cell currents, especially in the heterozygous state, but with no increased single-channel open probability and only a very small reduction in GqPCR sensitivity (Fig. S2). Thus, although the M2 helix clearly represents a hotspot for DDSA mutations, this variant is unlikely to underlie the phenotype observed in this patient and so also highlights a structural boundary for this specific hotspot of GoF variants.

### Equivalent disease-causing mutations in both TASK-1 and TASK-3

Due to their high degree of sequence homology (Fig. S1), TASK-1 and TASK-3 share many structural and functional properties, and we have previously shown that GoF variants found in DDSA produce identical functional effects when equivalent mutations are introduced at the same sites in TASK-3 channels (Sörmann et al., 2022).

Interestingly, the R131H DDSA variant reported above has also been identified as a disease-causing GoF variant in TASK-3, where it is associated with KIS (Cousin et al., 2022). This therefore suggests that a pathogenic GoF variant in TASK-1 also has the potential to cause disease if the equivalent mutation occurs in TASK-3 and vice-versa. Furthermore, we also identified an affected individual who fits the classical KIS developmental phenotype with a heterozygous L122V variant in *KCNK9*, demonstrating that this variant not only results in DDSA in TASK-1 (*KCNK3*) (Sörmann et al., 2022) but also the KIS neurodevelopmental phenotype if mutated in TASK-3 (*KCNK9*) (Table S1).

**Figure 3.**
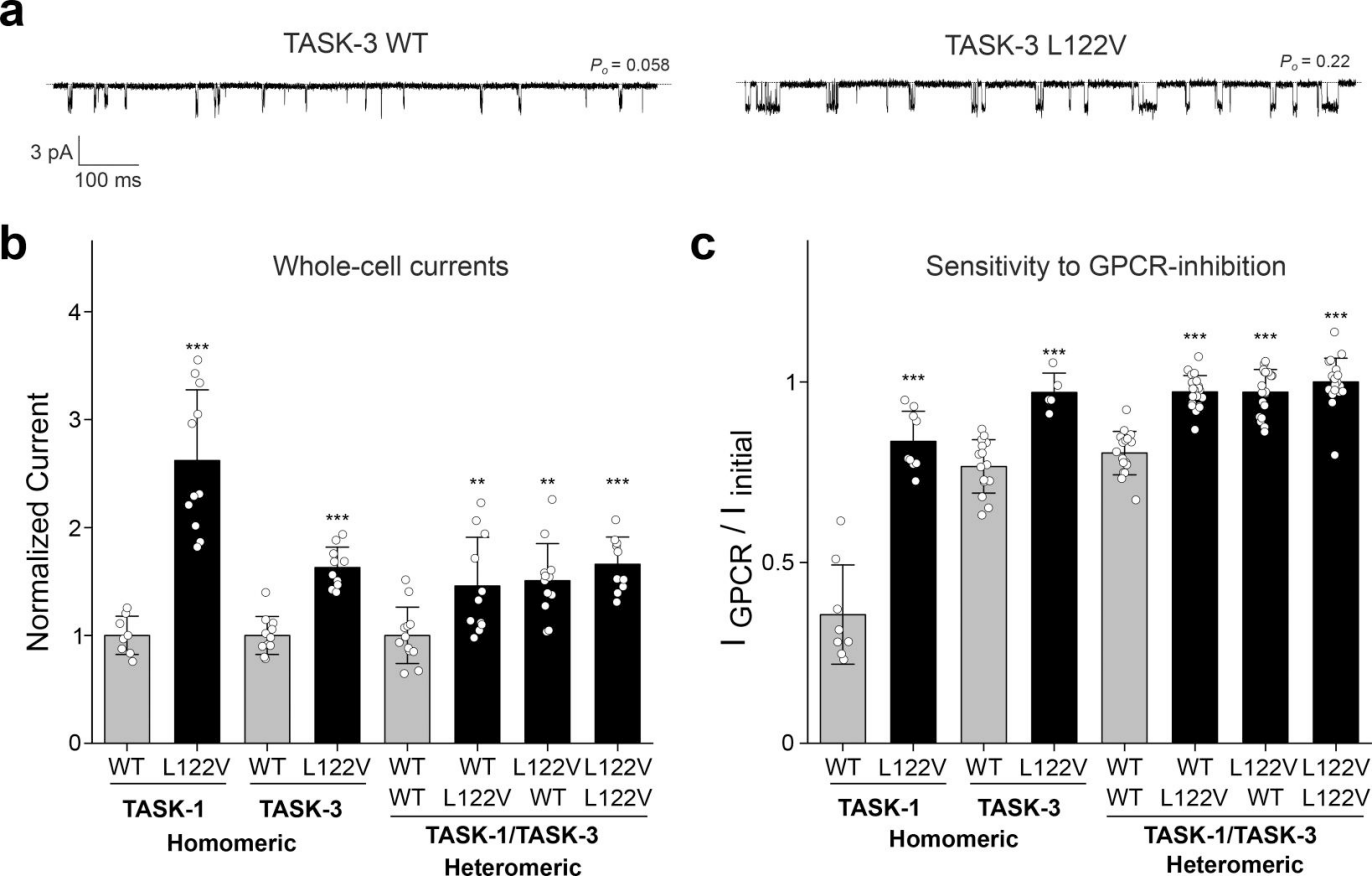
**Position-independent effect of a single L122V mutation in heteromeric TASK channels. (a)** Recordings of single channel currents at –160 mV showing the marked increase in *P*_*o*_ for TASK-3 L122V channels compared with WT. **(b)** Currents for homomeric and heteromeric WT and L122V TASK channels normalized to WT current at +50 mV. WT TASK-1 (*n* = 8), TASK-1 L122V (*n* = 11, P = 3.2 × 10^−6^), WT TASK-3 (*n* = 11), and TASK-3 L122V (*n* = 10, P = 2.0 × 10^−7^). For heteromeric TASK-1/TASK-3 channels: top row = TASK-1, bottom = TASK-3. WT-WT (*n* = 12), WT-L122V (*n* = 11, P = 0.0063), L122V-WT (*n* = 12, P = 0.0019), and L122V-L122V (*n* = 10, P = 0.00013). **(c)** Residual currents for homomeric and heteromeric TASK channels with different combinations of L122V mutations as indicated after GPCR-mediated inhibition. Residual currents measured after addition of 15 μM ATP. Labels same as shown in panel b. Homomeric: WT TASK-1 (*n* = 8), TASK-1 L122V (*n* = 9, P = 2.4 × 10^−7^), WT TASK-3 (*n* = 15), and TASK-3 L122V (*n* = 5, P = 6.5 × 10^−5^); heteromeric TASK-1/TASK-3: WT-WT (*n* = 17), WT-L122V (*n* = 23, P = 9.7 × 10^−14^), L122V-WT (*n* = 19, P = 6.7 × 10^-13^), and L122V-L122V (*n* = 20, P = 6.7 × 10^−16^). Data are presented as mean ± SD All mutant currents differ from their respective WT (one-way ANOVA with Dunnett’s multiple comparisons test). ns, not significant; ** = P < 0.01; *** = P < 0.001. One-way ANOVA with Tukey’s post hoc test used to show the position-independent effect of L122V in heteromeric TASK channels (all ns compared with each other P ≥ 0.2 in each case).

Mutations at this particular site (TM2.6) in the M2 helix have been shown to cause an activatory GoF effect in almost every known K2P channel (Ben Soussia et al., 2019), and in TASK-1 produce channels with increased channel open probability and reduced GqPCR-sensitivity (Sörmann et al., 2022). We therefore examined its effect in TASK-3 and found similar activatory effects that result from an increased single channel open probability (L122V TASK-3 *P*_*o*_ = 0.22 ± 0.06 (*n* = 8) compared with 0.058 ± 0.022 (*n* = 8) for WT TASK-3; Fig. 3). The fold activation produced by this mutation in homomeric TASK-3 is lower (∼4-fold) compared with its ∼10-fold effect on TASK-1 (Sörmann et al., 2022), but this is likely explained by the higher intrinsic *P*_*o*_ of WT TASK-3 compared with WT TASK-1 (Jouen-Tachoire et al., 2026).

The activatory effect of this L122V variant in TASK-3 suggests it may also have an equivalent effect on heteromeric TASK-1/TASK-3 channels, similar to the effect of the L122V variant in TASK-1 (Sörmann et al., 2022). We therefore examined the functional effects of the L122V mutation in heteromeric TASK-1/TASK-3 channels and found that whole-cell currents were increased ∼50% (Fig. 3 b) along with a markedly impaired sensitivity to GqPCR-mediated inhibition (Fig. 3 c) irrespective of which TASK subunit is mutated.

### Structural consequences of the TASK-3 L122V variant

The common activatory effect of TM2.6 mutations on K2P channel activity suggests a conserved gating mechanism in this region (Ben Soussia et al., 2019). In a previous study, we demonstrated that hydrophilic substitutions at this position reduced a hydrophobic barrier deep within the inner cavity of TWIK-1 (*KCNK1*) (Aryal et al., 2014), but it remains unclear whether this is the case in all other K2P channels.

**Figure 4.**
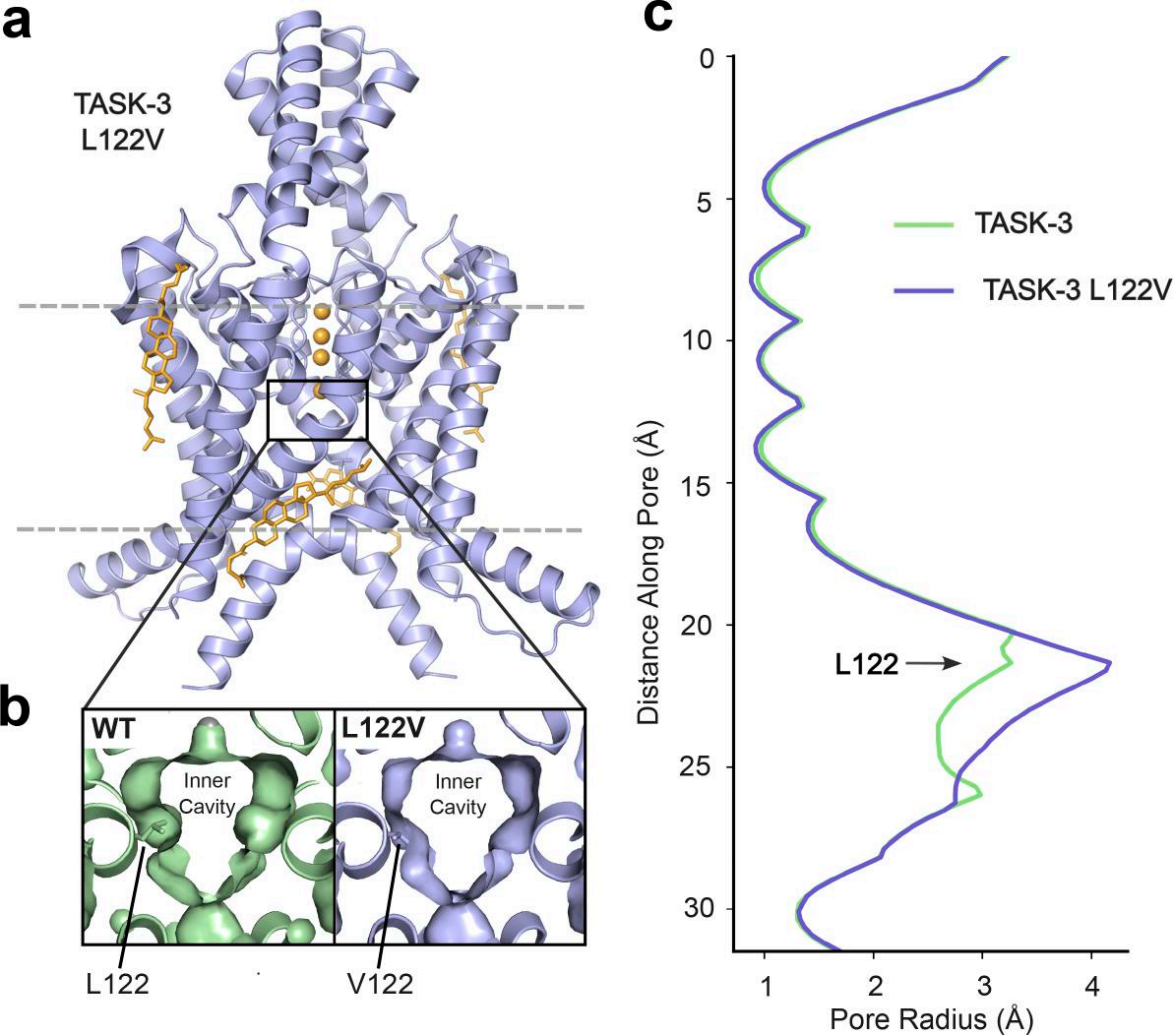
**Cryo-EM structure of the TASK-3 L122V variant reveals expansion of inner cavity below filter. (a)** Cartoon representation of the overall fold of the TASK-3 L122V mutant channel as determined by cryo-EM. K^+^ ions are shown as gold spheres and cholesterol hemisuccinate molecules as gold sticks. **(b)** Expanded view of the inner cavity at the site of the L122V mutation shown as a surface representation. The mutant L122V is shown in purple and WT TASK-3 in green. The side chains at position 122 are shown as sticks. Note the expanded inner cavity in the mutant channel. **(c)** Pore radius plot of WT TASK-3 and TASK-3 L122V. The expansion of the inner cavity below the filter at position 122 is indicated.

We have recently determined a structure of the WT TASK-3 channel by single-particle cryo-EM (Hall et al., 2025) and so used an identical method to determine the structure of this L122V variant to 2.8 Å resolution (Fig.4, Fig. S3, and Table S2). Comparison of this structure with WT TASK-3 reveals an identical overall fold, i.e., the channel is in the closed X-gate conformation with no obvious differences except within the inner cavity at the site of the mutation. In WT TASK-3, the closed X-gate forms a small inner cavity just below the filter with the L122 side chain lining this cavity (Fig. 4). In the L122V mutant structure, the smaller nature of the side chain increases the radius of the pore at this point from 3 to 4 Å and thus also increases the overall volume of the cavity immediately below the filter. This change is therefore perfectly situated to affect the interaction between K^+^ and water immediately prior to entry of K^+^ ions into the filter from within the inner cavity, as well as any hydrophobic gate which exists in this narrow cavity, but it may also have allosteric effects on the filter gate itself.

## Discussion

In this study, we have identified six further DDSA patients, with GoF variants in *KCNK3,* including three with novel GoF missense variants (F125S, Q126E, and R131H). This therefore markedly expands the global cohort of individuals with DDSA and reinforces the role that defective TASK-1 channels play in this unique neurodevelopmental disorder. We also report a novel TASK-3 variant (L122V) in an individual with KIS and have functionally and structurally characterized this mutant channel. These variants all share similar GoF effects on channel activity with a markedly reduced sensitivity to inhibition via GqPCR-coupled pathways. These GoF effects and their regulatory defects were also dominant in heterozygous channels containing one mutant subunit, as well as in heteromeric TASK-1/TASK-3 channels. Furthermore, we show that two of these pathogenic variants (R131H and L122V) are found in identical conserved positions in both *KCNK3* (TASK-1) and *KCNK9* (TASK-3) channels, and that their effects on heteromeric TASK channel activity appear the same, irrespective of which channel subunit contains the mutation.

Other examples also exist of equivalent pathogenic mutations in conserved domains of related channels that result in similar functional defects, yet different phenotypes, particularly within the superfamily of tetrameric cation channels. For example, the mutation of equivalent residues within the highly conserved DIV S3–S4 linker of the skeletal and cardiac muscle voltage-gated Na^+^ channels (*SCN4A* and *SCN5A*, respectively) produce similar effects on channel inactivation in these channels, yet result in different channelopathies (Mantegazza et al., 2021). Likewise, equivalent mutations within the positive charges of the conserved voltage sensor S4-helix in voltage-gated K^+^ channels also result in different channelopathies. In both cases, the unique phenotypes are due to the different spatiotemporal expression of these various channels, and so the distinct expression patterns for TASK-1 and TASK-3 will also heavily influence their phenotype. However, for the equivalent TASK channel mutations described here (e.g., L122V and R131H), the overlapping expression patterns of TASK-1 and TASK-3 channels and their ability to form physiologically relevant heteromeric channels is also important because any differences in the activity of the heteromeric channels that result from these mutations could be highly significant.

However, our results show that these equivalent mutations in either TASK-1 or TASK-3 have very similar effects on heteromeric channel activity. Given the very high degree of structural and functional conservation between TASK-1 and TASK-3 (Fig. S1), this is perhaps not surprising, but the markedly different disease phenotypes that result from these mutations therefore suggest it is defective homomeric TASK channels and their distinct spatiotemporal patterns of expression that may dominate the unique features of each disorder, especially within the developing CNS (Aller and Wisden, 2008). Such GoF mutations will not only dampen neuronal excitability, but it is well known that inappropriate spatiotemporal K^+^ channel expression can adversely affect the development of many cell types, especially neurons (D'Adamo et al., 2020).

Intriguingly, not all DDSA-associated variants (e.g., L239P) (Sörmann et al., 2022) result in a major increase in whole-cell currents even though they all increase channel *P*_*o*_. Indeed, the increase in currents for the three novel variants reported here, especially in the heterozygous state (Fig. 1 e), is relatively modest. Furthermore, in KIS some of the variants result in reduced TASK-3 channel activity (Cousin et al., 2022). However, in every case, these pathogenic mutants all share a marked reduction in their sensitivity to GqPCR-mediated inhibition. Therefore, in addition to any change in whole-cell currents, this loss of GqPCR sensitivity may represent a significant factor in defining their pathogenic phenotype. In particular, this may be especially important in the respiratory phenotype seen in all DDSA (and some KIS) individuals because GqPCR-mediated inhibition of TASK channels contributes to depolarization of cells in the carotid body as well as in pacemaker neurons in the brainstem important for sleep/wakefulness transition and respiratory rhythmogenesis. Abnormal respiratory control would therefore be expected to result from such defective channel regulation (Buckler et al., 2000; Trapp et al., 2008; Bayliss et al., 2015; Buckler, 2015).

The genomic imprinting reported for *KCNK9* and the mosaicism that arises from their *de novo* origin may also explain why only a subset of KIS patients are reported to have sleep apnea, compared with its defining presence in all known cases of DDSA (Luedi et al., 2007; Cooper et al., 2020; Cousin et al., 2022). However, there is also precedent in other genetic conditions involving defective K^+^ channels where, paradoxically, both LoF and GoF variants lead to neuronal hyperexcitability (Niday and Tzingounis, 2018) and/or neurodevelopmental disorders (D'Adamo et al., 2020). Consequently, much work remains to be done in order to understand this complexity.

Finally, our cryo-EM structure of the L122V variant at the TM2.6 site in TASK-3 also highlights how even subtle changes in structure can have dramatic functional consequences. Although the hydrophobicity of this side chain does not change, the slight increase in volume of the inner cavity just below the filter may be enough to affect a critical step in permeation, i.e., the dehydration of the K^+^ ion prior to its entry into the filter and to affect any hydrophobic gating mechanism in this region (Aryal et al., 2015). It is also consistent with the activatory effect of hydrophilic substitutions at this site (Aryal et al., 2014; Ben Soussia et al., 2019). Indeed, recent detailed simulations of K^+^ permeation in open state conformations of other K2P channels have shown that K^+^ permeation through the filter is tightly coupled to gating (Aldakul et al., 2025). This particular mutation therefore highlights the complexity of understanding the effect of missense variants on channel activity and their relationship to clinical phenotype, even when detailed functional assays and structural information are available.

In conclusion, we have characterized the functional and structural properties of a range of novel GoF missense disease-causing variants in TASK channels to expand the molecular and genetic basis of TASK channelopathies. These variants also provide insight into the relative contributions of homomeric versus heteromeric TASK channels in their respective disorders. Overall, our characterization of the structural and functional effects of these pathogenic variants has important implications for understanding genotype/phenotype correlations and also for future drug development studies involving both these rare channelopathies as well as other more common disease states involving TASK channels.

## Supplementary Material

Table S1 summarizes the phenotype and anonymized clinical details of the new DDSA and KIS patients reported in this study.

Table S2 shows cryo-EM data collection, refinement, and validation statistics.

## Data Availability

The data underlying Figs. 1, 2 and 3 are available in the published article and its online supplemental material. The atomic coordinates for the TASK-3 L122V model shown in Fig. 4 have been deposited in the PDB with accession number 28IZ. Coulomb potential maps have been deposited in the EMDB database, under the following accession code: EMDB-56538. Patient data included in Table S1 from the National Genomic Research Library (NGRL) used in this research are available within the secure Genomics England Research Environment. Access to NGRL data is restricted to adhere to consent requirements and protect participant privacy. Visit: https://www.genomicsengland.co.uk/research for more information on patient data access.
