# Supplementary material for: Equivalent gain-of-function variants in KCNK3 and KCNK9 and their contribution to distinct TASK K2P channelopathies
Source: J Gen Physiol. 2026 Aug 6;158(5):e202613989. doi: 10.1085/jgp.202613989 (PMC13446175; doi:10.1085/jgp.202613989)
Supplement: Supplementary file 1 [file jgp_202613989_tables1.docx]

**Supplementary Table S1**

| **Gene** | **TASK-1 (*KCNK3*)** | **TASK-1 (*KCNK3*)** | **TASK-1 (*KCNK3*)** | **TASK-1 (*KCNK3*)** | **TASK-1 (*KCNK3*)** | **TASK-1 (*KCNK3*)** | **TASK-3 (*KCNK9*)** |
| --- | --- | --- | --- | --- | --- | --- | --- |
| **Missense Variant** | **F125S** | **Q126E** | **R131H** | **N133S** | **N133S** | **L241F** | **L122V** |
| **Inheritance** | *De novo* | Unknown | *De novo* | *De novo* | *De novo* | *De novo* | Maternally inherited |
| **Sex** | Female | Male | Female | Female | Male | Male | Female |
| **Age** | 0-5yrs | 0-5yrs | 0-5yrs | 0-5yrs | 10-15yrs | 0-5yrs | 0-5yrs |
| **Sleep apnea** | Severe central sleep apnea  Mild obstructive sleep apnea | Severe central sleep apnea  Mild obstructive sleep apnea | Central sleep apnea | Severe central sleep apnea & obstructive sleep apnea | Central sleep apnea | Both obstructive and central, sleep apnea. Caffeine started, CPAP recommended | Abnormal sleep patterns with apneic episodes |
| **Developmental delay** | Moderate developmental and speech delay with generalized delayed of motor milestones. | Moderate developmental delay with generalized delayed of motor milestones. | Moderate developmental delay | Moderate developmental and speech delay | Delayed ability to sit; Delayed ability to walk | Moderate motor and speech delay. | Moderate developmental and speech delay |
| **Abnormalities of musculoskeletal system, limbs face and jaw** | Hypotonia, arthrogryposis, bilateral equinovarus talipes dolichocephaly, tent-shaped mouth, low-set ears | Hypotonia. Mild hypertelorism with epicanthal folds. Nevus flammeus over the forehead. | Hypotonia. Bilateral talipes | Hypotonia. Vertical talus | Hypotonia. Bilateral talipes. Abnormal facial shape & tongue. Bilateral ptosis | Hypotonia, severe head lag. Elongated face, tent-shaped mouth, big ears, low facial expressions | Hypotonia, torticollis. Narrow, downslanting palpebral fissures. Low-set posteriorly rotated ears with overfolded helices. Upturned nares. Open-mouth posture. Finger contractures, camptodactyly |
| **Abnormality of digestive system** | Feeding difficulties, nasogastric tube initially required | Feeding difficulties |  |  | Feeding difficulties | Difficulties with chewing and swallowing, constipation | Feeding difficulties. |
