# Supplementary material for: Equivalent gain-of-function variants in KCNK3 and KCNK9 and their contribution to distinct TASK K2P channelopathies
Source: J Gen Physiol. 2026 Aug 6;158(5):e202613989. doi: 10.1085/jgp.202613989 (PMC13446175; doi:10.1085/jgp.202613989)
Supplement: Supplementary file 2 [file jgp_202613989_tables2.docx]

**Supplementary Table S2**

**Cryo-EM data collection, refinement and validation statistics**

|  | **TASK-3 L122V**  **(EMDB- 56538)**  **(PDB 28IZ)** |
| --- | --- |
| **Data collection and processing** |  |
| Magnification | 105000 |
| Voltage (kV) | 300 |
| Electron exposure (e–/Å^2^) | 43.9 |
| Defocus range (μm) | 1.2-2.7 |
| Pixel size (Å) | 0.832 |
| Symmetry imposed | C2 |
| Initial particle images (no.) | 8381007 |
| Final particle images (no.) | 211355 |
| Map resolution (Å)  FSC threshold | 2.83 0.143 |
| Map resolution range (Å) | 2.43-33.5 |
|  |  |
| **Refinement** |  |
| Initial model used (PDB code) | 9g9v |
| Model resolution (Å)  FSC threshold | 2.8 0.143 |
| Model resolution range (Å) | 2.5-3.9 |
| Map sharpening *B* factor (Å^2^) | -130.3 |
| Model composition  Non-hydrogen atoms  Protein residues  Ligands | 4150  520  K^+^: 4, CHS: 6 |
| *B* factors (Å^2^)  Protein  Ligand | 83.94  92.25 |
| R.m.s. deviations  Bond lengths (Å)  Bond angles (°) | 0.0035  0.83 |
| Validation  MolProbity score  Clashscore  Poor rotamers (%) | 0.88  1.45  0 |
| Ramachandran plot  Favored (%)  Allowed (%)  Disallowed (%) | 98.06  1.94  0 |
